# Self-harm in prisons in England and Wales: an epidemiological study of prevalence, risk factors, clustering, and subsequent suicide

**DOI:** 10.1016/S0140-6736(13)62118-2

**Published:** 2014-03-29

**Authors:** Keith Hawton, Louise Linsell, Tunde Adeniji, Amir Sariaslan, Seena Fazel

**Affiliations:** a Centre for Suicide Research, Department of Psychiatry, University of Oxford, Oxford, UK; b Clinical Trials Unit, National Perinatal Epidemiology Unit, University of Oxford, Oxford, UK; c Equality, Rights and Decency Group, National Offender Management Service, Ministry of Justice, London, UK; d Department of Medical Epidemiology and Biostatistics, Karolinska Institute, Stockholm, Sweden; e Department of Psychiatry, University of Oxford, Oxford, UK

## Abstract

**Background:**

Self-harm and suicide are common in prisoners, yet robust information on the full extent and characteristics of people at risk of self-harm is scant. Furthermore, understanding how frequently self-harm is followed by suicide, and in which prisoners this progression is most likely to happen, is important. We did a case-control study of all prisoners in England and Wales to ascertain the prevalence of self-harm in this population, associated risk factors, clustering effects, and risk of subsequent suicide after self-harm.

**Methods:**

Records of self-harm incidents in all prisons in England and Wales were gathered routinely between January, 2004, and December, 2009. We did a case-control comparison of prisoners who self-harmed and those who did not between January, 2006, and December, 2009. We also used a Bayesian approach to look at clustering of people who self-harmed. Prisoners who self-harmed and subsequently died by suicide in prison were compared with other inmates who self-harmed.

**Findings:**

139 195 self-harm incidents were recorded in 26 510 individual prisoners between 2004 and 2009; 5–6% of male prisoners and 20–24% of female inmates self-harmed every year. Self-harm rates were more than ten times higher in female prisoners than in male inmates. Repetition of self-harm was common, particularly in women and teenage girls, in whom a subgroup of 102 prisoners accounted for 17 307 episodes. In both sexes, self-harm was associated with younger age, white ethnic origin, prison type, and a life sentence or being unsentenced; in female inmates, committing a violent offence against an individual was also a factor. Substantial evidence was noted of clustering in time and location of prisoners who self-harmed (adjusted intra-class correlation 0·15, 95% CI 0·11–0·18). 109 subsequent suicides in prison were reported in individuals who self-harmed; the risk was higher in those who self-harmed than in the general prison population, and more than half the deaths occurred within a month of self-harm. Risk factors for suicide after self-harm in male prisoners were older age and a previous self-harm incident of high or moderate lethality; in female inmates, a history of more than five self-harm incidents within a year was associated with subsequent suicide.

**Interpretation:**

The burden of self-harm in prisoners is substantial, particularly in women. Self-harm in prison is associated with subsequent suicide in this setting. Prevention and treatment of self-harm in prisoners is an essential component of suicide prevention in prisons.

**Funding:**

Wellcome Trust, National Institute for Health Research, National Offender Management Service, and Department of Health.

## Introduction

Suicide and self-harm are major issues in prisoners, yet they receive limited attention in national suicide prevention strategies.^1^, ^2^ Suicide rates in inmates of both sexes are far higher than in the general population in many countries.^3^ In England and Wales, standardised mortality ratios for suicide are five times higher in male prisoners^4^ and 20 times higher in female inmates^5^ than in general population controls. According to findings of a systematic review,^6^ about 50% of people who die by suicide in prison have a history of self-harm, which increases the odds of suicide in custody between six and 11 times.

In addition to being an important risk factor for prison suicide, self-harm is itself a major problem within prisons,^7^, ^8^ particularly because it is frequently repeated. However, information about prevalence, repetition, risk factors,^9^ and subsequent suicide in individuals who self-harm in custody is scarce. Furthermore, knowledge about whether clustering of self-harm takes place is important,^10^ because such phenomena might suggest contagion or specific environmental effects. To address these issues, we have undertaken a study of self-harm in the whole prison estate of England and Wales in 2004–09.

## Methods

### Study design

Our study consisted of four parts. First, we did a descriptive study using data obtained routinely for all self-harm episodes in all prisons in England and Wales between January, 2004, and December, 2009. Second, we undertook a case-control study of risk factors for self-harm. Third, we analysed clustering of self-harm. Finally, we did a comparative cohort study to identify risk of suicide after self-harm and associated risk factors. We received ethics approval from the National Offender Management Service.

### Procedures

For the descriptive study, we calculated prevalence of self-harm with two denominators: the average prison population for sentenced prisoners; and the number of receptions for unsentenced prisoners. The number of receptions has been suggested as a denominator for populations with substantial turnover.^11^ Annual prison population figures were based on the English and Welsh prison population, averaged over 12 months. Receptions were the number of unique individuals who entered prison for the individual years of the study.

Self-harm includes intentional self-poisoning or self-injury, irrespective of the degree of suicidal intent or underlying motive.^12^ In 2002, a mandatory reporting system for incidents of self-harm in prison was introduced in England and Wales. Prison officers must complete a paper form for every incident of self-harm, on which they record relevant details (appendix pp 1–2). From these data, we calculated the age distribution of individuals who self-harmed and categorised the number of incidents by type of prison and sentencing, and by the lethality of incidents and methods used to self-harm, for male and female prisoners separately. One of us (TA) estimated lethality of every self-harm incident on the basis of treatment outcome, an approach similar to other prison research.^13^ Incidents categorised as high lethality involve resuscitation in prison, an overnight stay in hospital, external hospitalisation on life support, or a combination of these. We defined medium lethality as an incident leading to external hospitalisation other than life support and low lethality as an episode not needing resuscitation or any external hospitalisation.

For the case-control analysis of risk factors for self-harm, we compared the characteristics of individuals with a history of self-harm (cases) with those of the rest of the prison population (controls). We restricted this analysis to the period January, 2006 to December, 2009, because control data were not available for earlier years. We defined cases as prisoners who self-harmed at least once during the period 2006–09; if the prisoner self-harmed on more than one occasion during this 4-year period, we used the details recorded at the first incident in this period. We excluded from our analyses any incidents of self-harm that were not linked to a unique prisoner number, when the unit of analysis was the individual prisoner. We selected the control population by taking a cross-section of the prison population on June 30 every year from 2006 to 2009, from inmates with no previous record of self-harm in prison. If the control prisoner appeared in more than one annual cohort, we extracted details from the first record in this 4-year period.

For the comparative cohort study, we ascertained the number of suicides in prison within the cohort of prisoners who self-harmed and identified risk factors that characterised inmates who subsequently died by suicide after a self-harm episode from those who did not. Self-inflicted deaths in prisons are identified on the basis of any death of an individual who has apparently taken his or her own life, irrespective of intent.^14^ This decision can be modified after a coroner's inquest.

### Statistical analysis

We investigated the following factors, separately by sex, to see whether they were associated with the risk of self-harm: age-group, ethnic origin, type of prison, sentence length, and previous violent offence against an individual (ascertained using all available records for every prisoner during the study period). We cross-tabulated every factor with self-harm status (yes or no) and tested associations in bivariate models with the χ^2^ test. We analysed all factors with a p value less than 0·05, by multivariate logistic regression.

We calculated the number of suicides while in custody among prisoners who self-harmed for the period 2004–09 and presented findings separately by sex. We looked at the association of the following factors with risk of suicide: age group, ethnic origin, nationality, type of prison, length of sentence, cell occupancy (single or with others), last method of self-harm, mean number of self-harm incidents per year, most lethal incident of self-harm, and previous violent offence against an individual. We tested associations with a bivariate analysis, using Fisher's exact test (because outcomes were infrequent), and did multivariate logistic regression to examine all factors, with a p value less than 0·05. We did sensitivity tests of SEs for model misspecification in the logistic regressions and re-ran them with a robust sandwich estimator, but differences were negligible.

To examine possible clustering of self-harm in time and by prison, we used a Bayesian estimation approach to produce intra-class correlation statistics. This method is appropriate for complex multilevel models^15^ (appendix pp 1–2).

### Role of the funding source

The sponsor of the study had no role in study design, data collection, data analysis, data interpretation, or writing of the report. The corresponding author had full access to all the data in the study and had final responsibility for the decision to submit for publication.

## Results

Between January, 2004, and December, 2009, 139 195 self-harm incidents involving 26 510 prisoners were recorded in England and Wales. Some individuals had events in more than one year; hence, the number of unique individuals who self-harmed (n=26 510) is less than the cumulative number who self-harmed each year (n=36 784; table 1). 6342 (5%) incidents were not linked to a unique prisoner number and were excluded from analyses of individuals.

**Table 1:** Incidents of self-harm by year and sex in all prisoners in England and Wales, 2004–09

	**2004**	**2005**	**2006**	**2007**	**2008**	**2009**
Prison population*						
Male inmates	70 209 (94%)	71 512 (94%)	73 680 (94%)	75 842 (95%)	78 158 (95%)	79 276 (95%)
Female inmates	4448 (6%)	4467 (6%)	4447 (6%)	4374 (5%)	4414 (5%)	4283 (5%)
Total	74 657 (100%)	75 979 (100%)	78 127 (100%)	80 216 (100%)	82 572 (100%)	83 559 (100%)
**Number of first receptions**†						
Male inmates	120 407 (91%)	119 783 (91%)	117 036 (91%)	114 034 (91%)	121 472 (91%)	114 833 (91%)
Female inmates	12 554 (9%)	12 275 (9%)	11 950 (9%)	11 847 (9%)	12 676 (9%)	11 044 (9%)
Total	132 961 (100%)	132 058 (100%)	128 986 (100%)	125 881 (100%)	134 148 (100%)	125 877 (100%)
**Incidents of self-harm**‡						
Male inmates	9849 (104 per 1000)	10 412 (113 per 1000)	11 886 (129 per 1000)	11 589 (123 per 1000)	12 211 (125 per 1000)	13 694 (122 per 1000)
Female inmates	9839 (1597 per 1000)	13 368 (2190 per 1000)	11 505 (1896 per 1000)	11 408 (1871 per 1000)	13 015 (2194 per 1000)	10 419 (1615 per 1000)
Total	19 688 (200 per 1000)	23 780 (247 per 1000)	23 391 (242 per 1000)	22 997 (232 per 1000)	25 226 (249 per 1000)	24 113 (208 per 1000)
**Individuals who self-harmed**§						
Male inmates	4193 (5%)	4405 (5%)	4652 (5%)	4976 (5%)	5148 (5%)	5340 (6%)
Female inmates	1274 (20%)	1371 (22%)	1325 (22%)	1352 (23%)	1392 (24%)	1356 (21%)
Total	5467 (6%)	5776 (6%)	5977 (6%)	6328 (6%)	6540 (6%)	6696 (7%)
**Ratio of incidents to individuals**						
Male inmates	2·2	2·2	2·5	2·3	2·3	2·5
Female inmates	7·3	9·4	8·6	8·2	9·2	7·4
Overall	3·4	3·9	3·8	3·5	3·8	3·5

Data are number of prisoners (% of total) or number of prisoners (weighted per 1000 prisoners). Weighted rates were calculated using the average prison population* as the denominator for sentenced prisoners and first receptions† as the denominator for prisoners on remand. Sentence data were missing for 10% of incidents, so these data are an underestimation of the true rates.

* Averaged over 12 months.

† Number of first receptions into custody for that year.

‡ Prevalence includes incidents for which prisoner number was not recorded.

§ Prevalence excludes incidents for which prisoner number was not recorded (5% of incidents).

The total number of incidents of self-harm per year rose from 19 688 in 2004 to 24 113 in 2009, and prevalence ranged from 200 to 249 per 1000 prisoners during the study period (table 1). About half the incidents were in female inmates, and the prevalence of incidents per 1000 prisoners was over tenfold higher in female than in male prisoners. An estimated 5–6% of male prisoners self-harmed every year compared with 20–24% of female inmates.

The incident ratios (number of incidents/number of individuals) show that male prisoners who self-harmed did so twice a year on average, whereas female inmates who self-harmed did so about eight times a year, with little variation in this pattern over the study period (table 1). However these average estimates were affected by a few individuals who frequently self-harmed. The median number of incidents per year among men and teenage boys who self-harmed was one (IQR 1–2, range 1–127), and the median number among women and adolescent girls was two (IQR 1–5, range 1–557). More than 100 self-harm incidents per year were recorded in two male prisoners and 102 female inmates. The women and teenage girls in this group accounted for 17 307 episodes (26% of the total number in female prisoners).

The age-distribution of inmates who self-harmed was similar for male and female prisoners (table 2) and did not vary much over the 6-year period from 2004 to 2009. Self-harm was more common in young inmates. People younger than 20 years typically accounted for 13% of the prison population, yet 23% of male inmates and 21% of female prisoners who self-harmed every year were in this age group.

**Table 2:** Risk factors associated with the first episode of self-harm among prisoners in England and Wales during 2006–09

	**Male prisoners**				**Female prisoners**			
	Self-harm (yes) (n=13 447)	Self-harm (no) (n=183 707)	Adjusted odds ratio (95% CI)*	p	Self-harm (yes) (n=3189)	Self-harm (no) (n=11 011)	Adjusted odds ratio (95% CI)*	p
**Age-group (years)**								
15–19	3054 (23%)	23 444 (13%)	1·00	..	657 (21%)	722 (7%)	1·00	..
20–29	5411 (40%)	74 342 (40%)	0·59 (0·55–0·64)	<0·0001	1329 (42%)	4197 (38%)	0·41 (0·36–0·47)	<0·0001
30–39	2954 (23%)	47 381 (26%)	0·50 (0·46–0·55)	<0·0001	767 (24%)	3459 (31%)	0·30 (0·26–0·35)	<0·0001
40–49	1280 (10%)	25 992 (14%)	0·38 (0·34–0·42)	<0·0001	319 (10%)	1942 (18%)	0·21 (0·17–0·25)	<0·0001
50–59	252 (2%)	8610 (5%)	0·21 (0·18–0·25)	<0·0001	75 (2%)	552 (5%)	0·18 (0·13–0·25)	<0·0001
≥60	72 (1%)	3938 (2%)	0·14 (0·10–0·18)	<0·0001	9 (<1%)	139 (1%)	0·10 (0·05–0·22)	<0·0001
**Ethnic origin**								
White	9954 (82%)	133 423 (73%)	1·00	..	2614 (87%)	7588 (69%)	1·00	..
Black	850 (7%)	26 864 (15%)	0·41 (0·38–0·45)	<0·0001	231 (8%)	2169 (20%)	0·39 (0·33–0·45)	<0·0001
Asian	759 (6%)	13 933 (8%)	0·68 (0·62–0·74)	<0·0001	42 (1%)	339 (3%)	0·39 (0·28–0·55)	<0·0001
Mixed	341 (3%)	5751 (3%)	0·68 (0·60–0·77)	<0·0001	94 (3%)	466 (4%)	0·61 (0·48–0·78)	<0·0001
Other	168 (1%)	3058 (2%)	0·59 (0·48–0·71)	<0·0001	23 (1%)	400 (4%)	0·18 (0·12–0·28)	<0·0001
**Type of prison (male)**†								
Local‡	7398 (59%)	96 276 (52%)	1·00	..	..	..	..	..
Category B or C, or IRC§	2327 (19%)	53 765 (29%)	0·64 (0·60–0·69)	<0·0001	..	..	..	..
Closed or juvenile	2483 (20%)	23 279 (13%)	0·91 (0·84–0·98)	0·01	..	..	..	..
Open	47 (<1%)	7717 (4%)	0·11 (0·08–0·15)	<0·0001	..	..	..	..
High security	247 (2%)	2670 (1%)	1·19 (1·00–1·41)	0·05	..	..	..	..
**Type of prison (female)**†								
Mixed local¶	..	..	..	..	294 (10%)	912 (8%)	1·00	..
Closed or juvenile	..	..	..	..	22 (1%)	242 (2%)	0·36 (0·22–0·59)	<0·0001
Female closed¶	..	..	..	..	495 (16%)	2227 (20%)	0·79 (0·65–0·95)	0·01
Female local	..	..	..	..	2128 (70%)	6424 (58%)	0·86 (0·73–1·00)	0·05
Female open	..	..	..	..	99 (3%)	1206 (11%)	0·52 (0·39–0·68)	<0·0001
**Length of sentence**								
<12 months	2108 (17%)	29 485 (16%)	1·00		721 (24%)	2759 (25%)	1·00	..
≥1 year to <4 years	2558 (21%)	60 358 (33%)	0·65 (0·61–0·69)	<0·0001	587 (19%)	3442 (31%)	0·73 (0·64–0·84)	<0·0001
≥4 years (excluding life)	1310 (11%)	36 230 (20%)	0·74 (0·68–0·80)	<0·0001	201 (7%)	1651 (15%)	0·71 (0·58–0·86)	0·0005
Life	689 (6%)	8719 (5%)	1·41 (1·26–1·57)	<0·0001	87 (3%)	157 (1%)	1·99 (1·46–2·71)	<0·0001
Unsentenced‖	5475 (45%)	48 859 (27%)	1·27 (1·19–1·35)	<0·0001	1422 (47%)	3000 (27%)	1·69 (1·50–1·90)	<0·0001
**Previous violent offence against a person**								
No	6602 (69%)	130 236 (73%)	1·00	..	1633 (65%)	8896 (83%)	1·00	..
Yes	2938 (31%)	48 830 (27%)	1·04 (0·99–1·09)	0·11	870 (35%)	1857 (17%)	1·87 (1·68–2·08)	<0·0001

* Adjusted for other risk factors listed in a multivariate logistic regression analysis.

† An additional 940 male prisoners and 152 female inmates self-harmed while under the care of the Prison Escort Management Service.

‡ Local prisons receive prisoners from court, who tend to be category B but can also be category A or unsentenced.

§ Immigration removal centre (IRC) is similar in security to a category C prison.

¶ Female closed prisons hold sentenced prisoners. Mixed prisons hold sentenced and unsentenced individuals.

‖ Unsentenced prisoners are remand prisoners (ie, those awaiting trial) and those who have been convicted but are awaiting sentencing.

The highest number of self-harm incidents over the 6-year study period was recorded in female local prisons (which receive prisoners from court; n=47 853 [34%]), followed by male category B local prisons (adult and young offenders combined; n=32 920 [24%]). Further details of the 6-year analysis are in the appendix (p 3; data for individuals who self-harmed for 4 years are presented in table 2). Most incidents occurred among sentenced prisoners (81 810 [66%] of 123 247 over the 6-year period), with the remainder in people on remand (awaiting trial) or before sentencing.

The most common methods of self-harm for both sexes were cutting and scratching, which were recorded for 45 141 (65%) of 69 634 incidents in male prisoners and 35 592 (51%) of 69 548 incidents in female inmates over the 6-year study period. The next most frequent methods used among men and teenage boys were poisoning, overdose, or swallowing objects not intended for ingestion (6079 [9%]), followed by hanging (5071 [7%]) and self-strangulation (3623 [5%]). The second most common method used by women and adolescent girls was self-strangulation (21 621 [31%]); other methods of self-harm—including impact injury, wound aggravation, ligature, suffocation, and biting—accounted for fewer than 5% of incidents.

Most self-harm incidents were categorised as low lethality. However, 6731 (10%) of 69 641 incidents by male prisoners were of medium or high lethality compared with 1753 (3%) of 69 554 in female inmates. Around 1% of incidents were of high lethality for both sexes and mostly entailed poisoning, overdose, or swallowing objects not intended for ingestion (25%), hanging (22%), self-strangulation (22%), and cutting (20%). Incidents of medium lethality were dominated by poisoning, overdose, or swallowing (43%) and cutting (39%).

Male prisoners were at heightened risk of self-harm if they were younger than 20 years, of white ethnic origin, in a high-security prison, and either had a life sentence or were unsentenced (table 2). Female prisoners were most at risk of self-harm if they were younger than 20 years, of white ethnic origin, in a mixed local prison, had a life sentence or were unsentenced, and had previously committed a violent offence against an individual (table 2).

The Bayesian estimation produced a crude intra-class correlation for prisons of 0·19 (95% CI 0·16–0·24), indicating that almost a fifth of the variation in self-harm behaviours could be attributed to the prison context. Adjustments for individual-level characteristics (sex, method, and lethality) and type of prison significantly improved model fit, and the final adjusted model accounted for 15% of the variation in self-harm behaviours (intra-class correlation 0·15, 95% CI 0·11–0·18). When we looked specifically at repeat self-harm, this clustering effect was reduced to 0·03 (0·02–0·04). Thus, the clustering effect mainly related to first episodes of self-harm.

109 suicides (411 per 100 000 average annual prison population) were recorded in custody among the 26 510 individuals who had self-harmed at any time between 2004 and 2009 (table 3); 95 deaths were in 21 104 male prisoners (450 per 100 000) and 14 suicides were in 5406 female inmates (259 per 100 000). The difference in the proportion of suicides between male and female prisoners who self-harmed was not substantial (relative risk 1·74, 95% CI 0·99–3·04, p=0·06). The mean annual rate of suicide among male inmates who self-harmed (334 per 100 000) was nearly double that of females who self-harmed (149 per 100 000). Estimated suicide rates in the prison population who did not self-harm were 79 per 100 000 in male inmates and 98 per 100 000 in female prisoners.

**Table 3:** Suicide in prison among individuals who self-harmed and for the whole prison population, by sex and year (2004–09)

	**Prisoners who self-harmed at any time in the study period (2004–09)**						**Prison population (2004–09)**					
	**Male inmates**			**Female inmates**			**Male inmates**			**Female inmates**		
	Suicides (n)	Prisoners who self-harmed (n)	Rate of suicide per 100 000	Suicides (n)	Prisoners who self-harmed (n)	Rate of suicide per 100 000	Suicides (n)	Prison population (n)	Rate of suicide per 100 000	Suicides (n)	Prison population (n)	Rate of suicide per 100 000
2004	13	4193	310	5	1274	392	83	70 209	118	13	4448	292
2005	13	4405	295	2	1371	146	74	71 512	103	4	4467	90
2006	14	4652	301	2	1325	151	64	73 680	87	3	4447	67
2007	22	4976	442	4	1352	296	84	75 842	111	8	4374	183
2008	19	5148	369	0	1392	0	60	78 158	77	1	4414	23
2009	14	5340	262	1	1356	74	58	79 276	73	3	4283	70
Annual mean	16	4785	334	2	1345	149	71	74 780	95	5	4406	113

The main method of suicide among prisoners who self-harmed was hanging (91 [83%] of 109 suicides). The most common self-harm method used in the last recorded incident before suicide was cutting or scratching (56% of suicides), followed by hanging (14% of suicides). More than half of suicides (58 [53%]) occurred within 1 month of the last self-harm episode, 17 (16%) were within 1–3 months, 14 (13%) were within 3–6 months, ten (9%) were within 6–9 months, and ten (9%) suicides happened more than a year after the last self-harm incident. All six suicides in prisoners younger than 20 years were within 3 months of the last self-harm incident.

Several factors were associated with suicide in bivariate analyses. In male prisoners, the factors that remained significant in multivariate analysis were older age (particularly men aged 30–49 years) and a previous self-harm incident of moderate or high lethality (table 4). Of 20 369 male inmates who self-harmed and for whom complete data were available for age and lethality, 527 had both risk factors and 12 died by suicide in prison, equivalent to a positive predictive value of 2·3% and sensitivity of 12·6%. 14 suicides were recorded in female inmates; therefore power to detect any differences was limited (table 5). Factors that were significant in multivariate analysis were a life sentence and more than five self-harm incidents per year. Of the 4967 women who self-harmed and for whom complete data were available for sentence and number of incidents, 57 had both risk factors and four of these died from suicide. When these two factors were included in a predictive model, the positive predictive value was 7·0% and sensitivity was 28·6%.

**Table 4:** Risk factors associated with suicide among male prisoners who self-harmed, 2006–09

		**Number of suicides among male prisoners who self-harmed**	**Suicide rate per 1000 among male prisoners who self-harmed (95% CI)**	Adjusted odds ratio (95% CI)*	**p**
Total		95/21 104	4·5 (3·6–5·5)	..	..
Age group (years; n=20 369)					
	15–19	4/3791	1·1 (0·3–2·7)	1·00	..
	20–29	30/8895	3·4 (2·3–4·8)	2·08 (0·72–6·03)	0·18
	30–39	35/5105	6·9 (4·8–9·5)	3·73 (1·29–10·8)	0·02
	40–49	23/2061	11·2 (7·1–16·7)	6·43 (2·15–19·2)	0·001
	≥50	3/517	5·8 (1·2–16·9)	3·36 (0·73–15·6)	0·12
High security prison (n=21 104)					
	No	87/20 573	4·2 (3·4–5·2)	1·00	..
	Yes	8/531	15·1 (6·5–29·5)	1·86 (0·74–4·68)	0·19
Length of sentence (n=18 712)					
	Not life	74/17 578	4·2 (3·3–5·3)	1·00	..
	Life	16/1134	14·1 (8·1–22·8)	1·67 (0·83–3·38)	0·15
Mean number of deliberate self-harm incidents per year (n=21 104)					
	≤1	47/13 887	3·4 (2·5–4·5)	1·00	..
	>1 to ≤5	42/6242	6·7 (4·9–9·1)	1·48 (0·93–2·38)	0·10
	>5	6/975	6·2 (2·3–13·3)	1·18 (0·41–3·38)	0·76
Previous violent offence against a person (n=15 553)					
	No	44/10 718	4·1 (3·0–5·5)	1·00	..
	Yes	36/4835	7·4 (5·2–10·3)	1·32 (0·81–2·13)	0·26
Most lethal incident of self-harm (n=21 104)					
	Mild	55/16 931	3·2 (2·4–4·2)	1·00	..
	Moderate or high	40/4173	9·6 (6·9–13·0)	2·66 (1·66–4·24)	<0·0001

* Adjusted for other risk factors listed in a multivariate logistic regression analysis (n=15 274). Because of the small number of events, the following variables were re-coded in the multivariate analysis: age categories 50–59 years and ≥60 years were collapsed; sentence length was re-coded as life versus not life; prison type was re-coded as high security versus not high security.

**Table 5:** Risk factors associated with suicide among female prisoners who self-harmed, 2006–09

		**Number of suicides among female prisoners who self-harmed**	**Suicide rate per 1000 among female prisoners who self-harmed (95% CI)**	Adjusted odds ratio (95% CI)*	**p**
Total		14/5406	2·6 (1·4 to 4·3)	..	..
Length of sentence (n=4967)					
	Not life	9/4791	1·9 (0·9–3·6)	1·00	..
	Life	5/176	28·4 (9·3–65·0)	10·6 (3·36–33·4)	<0·0001
Mean number of deliberate self-harm incidents per year (n=5406)					
	≤1	2/2553	0·8 (0·1–2·8)	1·00	..
	>1 to ≤5	3/1812	1·7 (0·3–4·8)	1·78 (0·29–10·8)	0·53
	>5	9/1041	8·6 (4·0–16·3)	8·56 (1·71–42·7)	0·009
Most lethal incident of self-harm (n=5406)					
	Mild	9/4361	1·9 (0·9–3·7)	1·00	..
	Moderate or high	5/775	6·5 (2·1–15·0)	1·15 (0·35–3·84)	0·82

* Adjusted for other risk factors listed in a multivariate logistic regression analysis (n=4967). Because of the small number of events, the following variables were re-coded in the multivariate analysis: age categories 50–59 years and ≥60 years were collapsed; sentence length was re-coded as life versus not life; prison type was re-coded as high security versus not high security.

## Discussion

We report a national study of 139 195 self-harm incidents in 26 510 individual prisoners. Previous reviews have focused on suicide in prison and on release,^16^, ^17^ and national initiatives—such as suicide prevention measures and drug treatment—have been implemented after published research highlighted high suicide rates in prisoners. Such programmes might have led to a recent reduction in suicide rates in inmates in England and Wales.^18^ By contrast, self-harm has received less attention, partly because of the scarcity of research (panel).

Panel **Research in context**

**Systematic review**

We retrieved two recent systematic reviews of self-harm in prisoners^9^, ^19^ and identified a systematic review of risk factors for suicide in prisoners.^6^ To supplement the review on suicide, we searched PubMed without any language restrictions between January, 2009, and June, 2013, using the following search terms: “suicid*” AND “prison*” OR “felon*” OR “jail*” OR “custod*” OR “remand*” OR “young offender* institution*” OR “youth offender* institution*” OR “penal”. We found three studies of risk factors for suicide in European prison populations, in which associations were reported with violent offences,^20^ increased cell occupancy,^21^ and self-harm.^22^ Furthermore, we identified one cross-sectional study of self-harm in prison that investigated correlates of lifetime suicidal ideation.^23^ Rates of suicide^3^, ^20^ and lifetime rates of self-harm^19^ are consistently higher in custody than in the general population, and self-harm is a major risk factor for suicide in prisoners.^6^, ^22^ Prevalence of self-harm in custody is 5–24%,^19^ with no study reporting on more than 500 prisoners who self-harmed. Risk factor research on self-harm in prison has been inconclusive with respect to age, sex, single-cell occupancy, being on remand (awaiting trial), violent index offence, previous custody, and duration in custody.^9^ However, some evidence suggests that white ethnic origin, previous self-harm, and mental disorders are risk factors for self-harm. We did not identify any studies of the risk of suicide after self-harm in a prison population or analysis of clustering for self-harm. Results of psychological interventions for self-harm in prisons have been based on small studies and are largely inconclusive.^19^

**Interpretation**

Our study is much larger than all previous studies of self-harm in prisons combined. We have provided some precision on rates of self-harm during custody and shown associations with female sex, young age, white ethnic origin, prison type, and a life sentence or being unsentenced; moreover, in women, we noted an association between rates of self-harm and having committed a violent offence against an individual. We estimated the risk of repetition, which was especially high in a subgroup of female inmates, and showed evidence of clustering in time and location of prisoners self-harming. The risk of subsequent suicide in prisoners who self-harmed is substantially greater than in the general prison population, and many deaths occurred shortly after an episode of self-harm. We also identified risk factors for suicide after self-harm, including older age and a previous self-harm incident of high or moderate lethality in male inmates; furthermore, in female prisoners, a history of more than five self-harm incidents within a year was associated with suicide. Our findings can assist in prevention of self-harm or suicide in prisons and are relevant for prevention of these events in other institutional settings. The method of data collection is one that could be adopted in prisons in other countries.

We have estimated that the annual prevalence of self-harm in custody is 5–6% in men and teenage boys and 20–24% in women and adolescent girls. This proportion is much higher than the 0·6% of the UK general population who reported self-harming in the preceding year.^24^ Repetition of self-harm was common, and a few female prisoners accounted for many episodes (102 inmates and 17 307 episodes). Cutting and scratching were the most frequent self-harm methods in both sexes; in female inmates, self-strangulation was common (31% of all episodes). Most incidents were of low lethality, particularly in female prisoners.

For both sexes, young age (<20 years), white ethnic origin, and either a life sentence or being unsentenced were associated with self-harm. In male prisoners, risk was also increased for those in high-security prison, whereas female inmates in mixed local prisons (with unsentenced and sentenced individuals) were at higher risk. On multivariate analyses, an association with self-harm was noted with young age and white ethnic origin; other factors were a life sentence (both sexes) and having committed a previous violent offence (female prisoners only). Some of these risk factors have been reported in previous smaller studies—eg, violent offending,^8^, ^23^ white ethnic origin, remand status, and female sex.^7^ Our study is based in one country with high rates of imprisonment; rates and risk factors need to be examined elsewhere, particularly in places with large prison populations. For example, in China, rates of suicidal ideation in prisoners are similar between sexes,^25^ and self-harm in US inmates differs by ethnic origin.^26^

Our study is the first, to our knowledge, to look at clustering of self-harm in prisoners.^10^ We recorded a substantial clustering effect—an adjusted intra-class correlation of 15%. This effect would be categorised as very large^27^ and compares with intra-class correlations in schoolchildren of 5% for health complaints and 20% for educational attainments.^28^ Such clustering for self-harm contrasts with prison suicides, for which estimated imitation rates of 1–11% have been reported.^29^

Self-harm in prison was clearly a risk factor for suicide in prison, particularly in male inmates. However, the absolute risk of suicide was fairly low. The main method of suicide was hanging, typically preceded by cutting or scratching. A change in the method of self-harm between non-fatal and fatal episodes is common in the general community.^30^ Suicide occurs fairly soon after a self-harm episode. In our study, factors associated independently with risk of suicide in male prisoners who self-harmed were age (particularly men aged 30–49 years) and previous self-harm of higher lethality. In female inmates, although statistical power was limited, greater risk was associated with a life sentence and multiple previous episodes of self-harm (>5 per year).

For prevention of self-harm and suicide in prisoners, raising staff awareness and further training are important.^31^ One key issue is whether individuals at risk can be identified at reception and appropriate preventive measures initiated. Factors associated with self-harm were mostly non-specific and had low predictive power for suicide. Future researchers should consider whether adding other factors, such as a history of self-harm outside prison or psychiatric morbidity,^32^, ^33^ can improve screening. In terms of management, the individual factors we identified are largely unmodifiable; future investigations should focus on psychosocial characteristics amenable to intervention, such as depression, bereavement, self-esteem, and impulsivity.^34^ Our findings on clustering are potentially important because they suggest that prison-level changes in self-harm management might affect self-harm rates. Moreover, clustering was substantially more pronounced for prisoners self-harming once, rather than being a determinant of repetition. Therefore, this finding suggests that the response to self-harm should extend beyond the individual prisoner to other inmates in the same wing or prison who could be at risk.

Once an individual has self-harmed, consideration should be given to measures to prevent escalation, particularly because female prisoners who self-harm will on average have eight episodes a year. Introduction of a case-management approach for suicide risk in English and Welsh prisons—known as ACCT (Assessment, Care in Custody and Teamwork)—might increase the numbers of people receiving primary mental health care.^18^ Additional mental health input to this process, which is currently led by prison officers and does not necessarily include mental health professionals, is needed. Having prison staff lead this treatment is appropriate, but in more serious and repetitive cases, medical and psychological treatment should be considered. On the basis of current evidence from other settings, suicide risk management for prisoners should include psychosocial assessment, brief psychological treatment after an episode, and—for frequent self-harmers—modified dialectical behavioural therapy.^12^ These interventions require evaluation in prisoners, with trials of an adequate size.^9^, ^12^ Because prisoners who self-harm usually have several psychiatric comorbidities and psychosocial difficulties,^34^, ^35^ interventions might need to be more complex than in the general community, be multidisciplinary, and include speciality input. Restriction of access to means for self-harm is also important, similar to suicide prevention in psychiatric inpatients.^36^ Overall, our findings are consistent with calls for greater health-care involvement in the management and prevention of self-harm in prisons.^37^ However, the institutional challenges of improving prisoners' health care must be considered.^16^ One major challenge is negative attitudes of prison officers and health-care staff, and addressing these beliefs should be part of any self-harm strategy.^31^ Other solutions include closer involvement of academic medicine, regional networks, and international organisations, and increased legislation about prison health care. Underscoring all these points is the need for further spending on prison health care and ensuring that the proportional allocation of mental health funding is at least equivalent to that in the community.^38^

Our study had several limitations. First, the quality of data entry into the prison reporting system might vary by establishment; the number of unrecorded incidents is unknown and 5% of incidents did not have prisoner identifiers. Thus, data for recorded incidents are accurate (the annual rate of self-harm was fairly steady) but the numbers of individuals involved are estimates at the lower limit. Second, we were only able to include a limited number of variables in risk factor analyses. Excluded factors of interest were: time since first reception, previous offending, medication, and being identified as at risk. No information was available for suicidal intent,^39^ brain injury,^23^ psychiatric disorders,^32^, ^33^ personality factors, family history, and cell occupancy (in controls), which are associated with prisoner self-harm. Third, because risk factor information for suicide was recorded at the last self-harm episode, which was sometimes many months before death occurred, other relevant factors might not have been identified. We were unable to link the data to suicides in the high-risk post-release period.^17^ Finally, differences between prison stay of included individuals mean that periods at risk for self-harm and suicide will have varied considerably. Ideally, person-years at risk should be reported, a challenge for prison services worldwide, and this information would allow for more accurate estimates of hazards for adverse outcomes in custody and on release.

In conclusion, the burden of self-harm in prisoners is substantial, affecting 5–6% of male prisoners and 20–24% of female inmates every year. Repetition of self-harm is common, particularly in a subgroup of female prisoners. Evidence of clustering of self-harm episodes suggests that contagion might contribute to rates. Moreover, our results indicate that prevention of suicide in prisons should include a focus on inmates who are self-harming. We have identified some factors that could help target prisoners at risk; however, the fairly low base rate of suicide even in this high-risk group, and the paucity of risk factors, probably means that all prisoners who are self-harming should be regarded as at risk, with special focus on women who repeatedly self-harm.
