# Supplementary material for: Self-harm in prisons in England and Wales: an epidemiological study of prevalence, risk factors, clustering, and subsequent suicide
Source: Lancet. 2014 Mar 29;383(9923):1147–54. doi: 10.1016/S0140-6736(13)62118-2 (PMC3978651; doi:10.1016/S0140-6736(13)62118-2)
Supplement: Supplementary appendix [file mmc1.pdf]

THE LANCET

Supplementary appendix

This appendix formed part of the original submission and has been peer reviewed. We post it as supplied by the authors.

Supplement to: Hawton K, Linsell L, Adeniji T, Sariaslan A, Fazel S. Self-harm in prisons in England and Wales: an epidemiological study of prevalence, risk factors, clustering, and subsequent suicide. *Lancet* 2013; published online Dec 16. http://dx.doi.org/10.1016/S0140-6736(13)62118-2.

Details on recording of self-harm incidents

Date and location, cell type and occupancy, details of the method of self-harm used (including ligature point and type, location of any injury, type of instrument used, and what was swallowed), treatment received (including whether resuscitation was given, admission to hospital, length of hospital stay - overnight or over 24 hours, whether life support was needed), and free text to describe in more detail the incident. All prison officers are trained in the use of these forms. Prisoner name is linked with the Inmate Information System, a total register of all prisoners, to gather information on age, gender, ethnic group, nationality, medication, type of sentence, and type of crime committed. The information collated on each individual is entered on to the Prison Service Central Incident Reporting System database by prison administration staff, specifically trained to do so, downloaded monthly by the Offender Safety, Rights and Responsibilities Group, who check for inaccuracy and clarify missing information, and stored in a separate self-harm database for analysis. The data can be aggregated according to individuals using unique prison numbers in conjunction with names and dates of birth. As there were increased reporting rates over 2002-2003, we started data collection from 2004, when the monitoring forms were used throughout the prison estate. In addition, we manually investigated missing variables on prison number, age, gender and methods used on all reported incidents by searching free text descriptions of these incidents included on the forms, cross-checking with other covariates, and linkage with the Inmate Information System if there were other times when a particular individual was in custody.

Methods used to analyse clustering

We examined clustering of prisoners who self-harmed by time and location by estimating prison intra-class correlations (ICCs), measuring the degree to which prisoners within the same prison settings were similar to one another in terms of their propensity to self-harm, and by generalized linear mixed effects models (GLLMs) with binary outcomes.[1] The ICCs were calculated using the latent variable method, a standard approach in the field.[2] To account for prisoners transferring between different prison settings over time and thus improving the accuracy of the estimated ICCs, we adopted a "multiple membership" approach[3] where the prisoners were allowed to be members of multiple clusters (i.e. prisons) weighted by the proportion of time (in years) they had spent in each prison setting. The latter model extension required a Bayesian inference approach using Markov Chain Monte Carlo (MCMC) estimation techniques with non-informative priors[4] (and specified the models to run for 50,000 simulations with a burn-in period of 10,000 iterations and a thinning factor of 1). An assessment of model improvements was made using the Bayesian Deviance Information Criterion (DIC), where a decrease of 10 units is considered to be a significant improvement in model fit.[5] All models were fitted in MLwiN 2.26[6] through the runmlwin[7] interface based in Stata 12.1 IC.[8] These models were underpowered to conduct sensitivity analyses by gender, specific prison category, method and lethality.

Appendix references

1. Pinheiro JC, Bates DM. Mixed-effects models in S and S-Plus. New York: Springer-Verlag; 2000.
2. Larsen K, Merlo J. Appropriate assessment of neighborhood effects on individual health: Integrating random and fixed effects in multilevel logistic regression. *Am J Epidemiol* 2005; **161**: 81-8.
3. Browne WJ, Goldstein H, Rasbash J. Multiple membership multiple classification (MMMC) models. *Stat Modelling* 2001; **1**: 103-24.
4. Browne WJ. MCMC estimation in MLwiN. Bristol, UK: Centre for Multilevel Modelling, University of Bristol; 2009.
5. Spiegelhalter DJ, Best NG, Carlin BP, Van Der Linde A. Bayesian measures of model complexity and fit. *J R Stat Soc Series B Stat Methodol* 2002; **64**: 583-616.
6. Rasbash J, Browne W, Goldstein H, et al. A user's guide to MLwiN. London: Centre for Multilevel Modelling, Institute of Education, University of London; 2002.
7. Leckie G, Charlton C. RUNMLWIN: Stata module to run the MLwiN multilevel modelling software from within Stata. Statistical Software Components. Chestnut Hill, MA: Boston College Department of Economics; 2012.
8. StataCorp. Stata Statistical Software: Release 12. College Station, Texas: StataCorp LP; 2011.

Self-harm by prison establishment

Next in frequency were female closed prisons (those that hold sentenced prisoners (13,989 [10%]), followed by young offender (18-21 yrs) and juvenile institutions (under 18yrs) (12,352 [9%]), and then male category C prisons (holding sentenced prisoners) (9053 [6.5%]). In all other types of prison, including high security (Category A high risk prisoners), the number of incidents accounted for 5% or less of the total, and 2327 (2%) occurred while in the care of the Prison Escort Management Service (whilst in transit from court or inter-prison transfers). The proportions of incidents in each type of establishment were fairly constant, except in female closed prisons (holding sentenced women) where they decreased from 18% in 2004 to 7% in 2009. This is likely to be due to service re-configuration; some female prisons changed to male local prisons during 2004-2006, hence these cases of female self-harm appeared in different categories of prison from 2007 onwards. The percentage of incidents which occurred in female local prisons peaked in 2008 at 40% and dropped back to 29% in 2009.
